# Loss of tyrosine 211 phosphorylation of proliferating cell nuclear antigen (PCNA) enhances postnatal mammary gland development

**DOI:** 10.37796/2211-8039.1462

**Published:** 2024-09-01

**Authors:** Yi-Chun Shen, You-Zhe Lin, Wan-Rong Wu, Pei-Le Lin, Chien-Ching Liao, Feng-Chi Chung, Chia-Yun Chen, Ching-Yu Weng, Shao-Chun Wang

**Affiliations:** a Center for Molecular Medicine, China Medical University Hospital, Taichung 404327, Taiwan; b Cancer Biology and Precision Therapeutics Center, China Medical University, Taichung 406040, Taiwan; c Graduate Institute of Biomedical Sciences, College of Medicine, China Medical University, Taichung 406040, Taiwan; d Cancer Biology and Drug Discovery Ph.D. Program, China Medical University, Taichung 406040, Taiwan; e Department of General Medicine, China Medical University Hospital, Taichung 404327, Taiwan; f Department of General Medicine Kaohsiung Chang Gung Memorial Hospital, Kaohsiung 833401, Taiwan; g Department of Biotechnology, Asia University, Taichung 413305, Taiwan; h Department of Cancer Biology, University of Cincinnati, Cincinnati, OH 45267, USA; i School of Medicine, China Medical University, Taichung 406040, Taiwan

**Keywords:** Mammary gland, PCNA, Phosphorylation

## Abstract

The intricately orchestrated progression of mammary tissue development involves the precise coordination of gland differentiation and cellular proliferation. Nevertheless, the understanding of the role and regulatory mechanisms governing the DNA replication machinery in mammary gland development remains limited. Given the essential role of DNA replication in the viability of living cells, any genetic disturbance to its replicative function, in any form, will impede organ development. This circumstance poses a technical challenge in elucidating the potential function of cell proliferation in mammary morphogenesis. PCNA is crucial in DNA replication, playing a pivotal role in the development of complete eukaryotic organisms. The phosphorylation of PCNA at tyrosine 211 (Y211) has been demonstrated to play a significant role in supporting replication forks and, consequently, cell proliferation. Therefore, the utilization of a knock-in mouse model, wherein the Y211 residue of PCNA is replaced with phenylalanine (211F), presents an opportunity to evaluate the impact of reduced cell proliferation potential on mammary gland development. Interestingly, the lack of Y211 phosphorylation did not significantly impact the rates of proliferation or cell death in the mammary gland. In contrast, the absence of Y211PCNA led to an increased, rather than reduced, growth of the mammary gland. This was evident in assessments of gland length and the number of terminal end buds (TEBs) in both postnatal and virgin mammary glands. Notably, this observation correlated with an elevation in tissue stemness within the 211F glands compared to the WT glands. Additionally, it was consistent with the greater body weight gains observed in 211F pups compared to WT pups during the weaning period. Our findings unveil an unexpected aspect that may carry significance for mammary development. This newfound is associated with the regulation of a central component within the DNA replication machinery, providing insights into the intricate interplay governing mammary tissue expansion.

## 1. Introduction

The postnatal maturation of mammary glands is a distinctive biological phenomenon when compared to other organs. The substantial and extensive remodeling of mammary tissues during puberty and pregnancy, influenced by hormonal and environmental cues, has been extensively documented. However, our comprehension of the early development of quiescent virgin mammary glands is still limited. Murine rudimentary mammary glands emerge through the formation of terminal end buds (TEBs), analogous to terminal ductal lobular units (TDLUs) in humans [1–4]. Consisting of multiple layers of cuboidal epithelial cells at the ductal termini, these TEBs extend into adipose pads containing adipocytes, stromal cells, and extracellular matrix. Eventually, they branch to establish a ductal network. This progression, termed branching morphogenesis [5], pauses until further stimulation during puberty or pregnancy induced by circulating hormones.

Within the mammary ducts, two principal lineages coexist: the inner luminal layer and the outer myoepithelial layer [4]. Genetic lineage-tracing studies have established a hierarchical lineage of stem and progenitor cells within these ductal layers in postnatal mammary ductal network development and maintenance [6,7]. Hence, both proliferation and differentiation are cohesively integrated into the developmental program of mammary glands. However, how the conventional DNA replication machinery is involved in developing mammary glands remains incompletely understood. This poses an intriguing issue to address because the functional attenuation of proliferative functions, such as DNA polymerase or the associated protein complex required for DNA synthesis, will lead to the inhibition of cell viability and, consequently, the loss of organ differentiation.

The proliferative protein PCNA, well-known for its versatile involvement in DNA replication and repair [8,9], functions as a trimeric ring enveloping the DNA double helix. It serves as a foundational platform crucial for DNA synthesis during cell proliferation and repairing processes of DNA damages such as double-strand break repair, mismatch repair, nucleotide excision repair, and base excision repair. PCNA also plays a crucial role in restoring chromatin post-replication, reinstating the epigenetic memory of the genome [1].

The intricate functionality of PCNA is delicately regulated by post-translational modifications. For instance, ubiquitylation of PCNA at lysine 164 serves as a signal directing the choice between the error-prone translation DNA synthesis pathway and the precision-oriented damage avoidance mechanism via homologous recombination [8,10]. Additionally, PCNA can undergo phosphorylation at specific tyrosine residues, exemplified by the phosphorylation of tyrosine 211 (Y211) (pY211-PCNA) through growth factor receptor signaling [11,12]. This phosphorylation stabilizes PCNA on chromatin, contributing to growth progression in cancer cells. In this regard, a genetic mouse model was instrumental in revealing the functional role of pY211-PCNA in tumor advancement. This model replaced the tyrosine 211 residue of PCNA with phenylalanine (F), a structurally analogous residue lacking the hydroxyl group on its aromatic ring and thus devoid of phosphorylation [13]. Intriguingly, mice homozygous for this 211F mutation developed normally. However, when introduced into the genetic background of MMTV-PyMT mice, expressing the oncoprotein polyoma virus middle T antigen (PyMT) driven by the mammary gland-specific enhancer/promoter of the mouse mammary tumor virus (MMTV), they displayed the ability to form spontaneous mammary gland tumors, akin to wild-type (WT) mice [14]. Extensive genetic and cell biology investigations focusing on these 211F/PCNA “knock-in” mice concluded that pY211-PCNA contributes to the stemness and tumor-initiating potential of primary cancer cells [13,15]. These results collectively demonstrate the impact of pY211-PCNA in mammary tumorigenesis, but the role of Y211 phosphorylation in the development of normal mammary gland has remained unexplored.

We speculated that, given the well-documented proliferative role of phospho-Y211 PCNA in vegetative cells, PCNA Y211 phosphorylation should also be required for mammary gland development. To test this hypothesis, we leverage the established 211F/PCNA knock-in mouse model to investigate potential effects on mammary gland progression. Surprisingly, our findings unveil an entirely unforeseen aspect of PCNA’s contribution to the normal development of mammary glands.

## 2. Methods and materials

### 2.1. Animal models

The generation of the Y211F PCNA knock-in mice has been reported [13]. Briefly, homologous recombination in mouse embryonic stem cells was referenced to mouse chromosome 2 (Ensembl database). The 5′ (6.3 kb) and 3′ (3.0 kb) homology arms were PCR amplified, confirmed by restriction digestion, and sequencing. The Y211F mutation (TAC to TTC) was introduced into the 3′ arm via PCR-based mutagenesis. The final vector contained Frt sequences, a Neo expression cassette (for positive selection), and a DTA cassette (for negative selection). The NotI linearized vector was electroporated into FVB ES cells, followed by neomycin/G418 selection. Clones were validated by PCR and Southern analysis. Neo-deleted clones were confirmed by G418 sensitivity and PCR. Injections into C57BL/6NTac blastocysts produced high-percentage male chimeras, which were then mated with wild-type FVB/N females. Heterozygotes were confirmed by genotyping PCR and sequencing. Animals were acclimatized in the room with controlled a 12 h light/dark cycle (lights on at 8:00 A.M.), temperature (21–23 °C), and air humidity (45–70%). Female mice aged eight to ten weeks were used for whole mount analysis and immunohistochemical staining of mammary glands. The litters were weaned, and the body weighed three weeks after birth. All of the animal breeding and experiments were conducted following the animal protocols approved by the Institutional Animal Care and Use Committee of the China Medical University, in addition to adhering to all relevant guidelines and regulations, including the ARRIVE guidelines.

### 2.2. Whole mount analysis of mammary glands

The 4th or 9th pair of mammary glands were removed from eight- and ten-week-old mice, and blunt forceps were used to spread and flatten them onto glass slides. Then, the slides were fixed in Kahle’s buffer overnight. After fixation, the tissue was washed in 70% ethanol for 15 min, followed by a 5-min rinse with distilled water, and then stained with Carmine alum overnight. The stained tissue was gradually dehydrated in a series of alcohol solutions (70%, 95%, and 100%; 15 min each). It was then cleared in xylene and mounted with a coverslip for fixation. After mounting to the glass slides, the tissue is analyzed under a dissecting microscope, and TEB (terminal end buds) and ductal length are counted. The ductal length is measured by the distance from the lymph node to the furthest terminal bud. The total numbers of TEBs in the whole mount glands were determined by direct counting. These TEBs were identified based on their morphological characteristics as budding structures with a diameter of 100 μm or greater. The cumulative count of TEBs from all the boxes within a gland was then tallied to establish the average TEB count per area.

### 2.3. Immunohistochemical staining

Section slides of formalin-fixed paraffin-embedded mammary gland tissues (5 μm) were deparaffinized by heating the slides at 65 °C for 20 min, followed by two rounds of 5 min incubation in xylene. The sections then rinsed for 5 min in pure ethanol, followed by 5-min rinses in 95% ethanol twice, and then a 5 min immersion in 75% ethanol, finally with a 5-min immersion in 50% ethanol. The dehydrated slides were then subjected to two rounds of 5-min washes by water. Antigen retrieval was performed by heating the slides in 1 × citrate buffer (CBB500, ScyTek) in a steamer for 15 min. The slides were allowed to cool down and washed three times with TBST (0.05% Tween 20 in TBS). The endogenous peroxidase activity was quenched by treating the slides with 3% H_2_O_2_ in double-distilled water for 10 min, then washed three times with TBST. To block the endogenous biotin present in the tissue sections, the slides were incubated with Antibody Diluent/Blocking buffer (ARD1001A, PerkinElmer) for 10 min. Primary antibodies, including cleaved caspase-3 (CST9661, Cell Signaling), Ki67 (CST9129, Cell Signaling), E-cadherin (CST3195, Cell Signaling), anti-αSMA (ab7817, Abcam), Cytokeratin 8 (ab53280, Abcam), and Keratin 14 (905303, BioLegend), were diluted using Antibody Diluent/Blocking buffer (ARD1001A, PerkinElmer), and then applied overnight. Subsequently, the primary antibodies were removed by thorough washing with TBST. The secondary antibodies, biotinylated goat anti-rat and Opal Polymer HRP Ms + Rb (ARH1001A, PerkinElmer), were diluted in the Antibody Diluent/Blocking buffer (ARD1001A, PerkinElmer) and applied to the tissues and incubated at room temperature for 1 h. Subsequently, the secondary antibodies were removed by thorough washing with TBST. The Opal 520/570/690 reagents (FP1487001KT, FP1488001KT, FP1497001K, PerkinElmer T) were diluted in 1 × Amplification Diluent 1 (FP1498A, PerkinElmer), and then dispensed onto the slides. The slides were incubated for 5 min and the reagent was subsequently removed by thorough washing with TBST. The slides were mounted with DAPI and examined using a confocal microscope. The IHC staining were quantified using ImageJ. Within the ImageJ image processing pipeline, fluorescence images underwent initialization and were subsequently subjected to an 8-bit conversion, to facilitate subsequent quantitative analysis. A meticulously defined threshold was systematically applied to enhance the delineation of targeted regions. Leveraging the Particle Analysis tool, the essential parameters such as area, intensity, and particle count were rigorously quantified in a systematic manner.

### 2.4. Flow cytometry analysis of mammary glands

Mammary glands excised from four WT and four Y211F FVB mice were rinsed with serum-free media and cut into small chunks. The tissue blocks were disintegrated into single cells by GentleMACS in Hank’s balanced salt solution (HBSS) containing 20 ug/ml DNase I, 2 mg/mL collagenase IV, and 5 mM MgCl2. Red blood cells were cleared by lysis with the ACK RBC lysis buffer. After being washed with PBS, re-suspended cells were collected and subjected to FACS analysis. The ALDH activity was determined by the ALDEFLUOR™ Kit (#01700, Stem Cell Technologies) according to the manufacturer’s instructions. Cells were incubated with activated ALDEFLUOR substrate for 60 min at 37 °C, and control samples were incubated with activated ALDEFLUOR substrate in the presence of the ALDH inhibitor DEAB (diethylaminobenzaldehyde), which is used to control for background fluorescence. For analyzing the membrane expression of the cluster of differentiation (CD) markers, clustered cells were excluded by gating with FSC-H and FSC-A (P1), cell debris were excluded by gating with FSC-A and SSC-A (P2), dead cells were excluded with 7-AAD (420403, BioLegend) staining (P3), and Sca-1 lineage positive cells were excluded with anti-mouse Sca-1-FITC (160907, BioLegend) staining. The remaining subpopulations were analyzed by anti-mouse CD29-PE (102207, BioLegend) and anti-mouse CD24APC (138505, BioLegend) staining.

## 3. Results and discussion

**Fig. 1:**
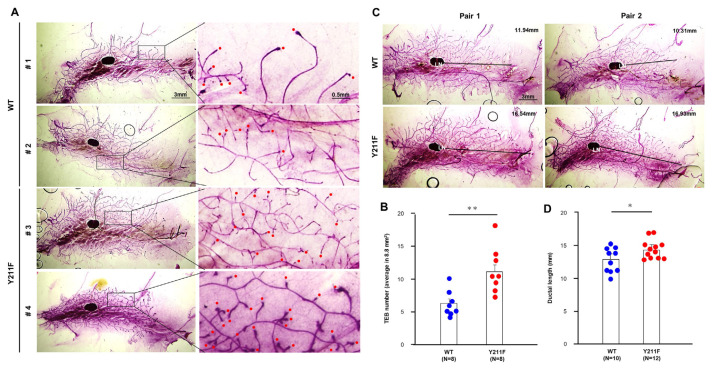
Increased gland extension of the mammary glands derived from the 211F mice. Whole-mount staining was performed on the mammary glands of eight to ten-week-old WT and 211F FVB mice. (A) Schematic presentation of counting the TEBs which are highlighted by red dots. (B) Quantification of TEB number. *, p < 0.05. (C) Schematic presentation of ductal length assessment. Each ductal length was measured from the lymph node (LN) to the farthest terminal bud. (D) Quantification of ductal length. **, p < 0.01.

To assess gland growth activity, we performed a quantitative analysis of the terminal end buds (TEBs) in the whole-mounts of mammary glands (Fig. 1A). Remarkably, the mammary glands from the 211F mice displayed a significantly higher number of TEBs compared to the wild-type (WT) glands (Fig. 1B). When examining gland sizes, we measured the distances from the lymph node to the farthest terminal bud and compared these measurements between the WT and 211F glands (Fig. 1C). The results of this analysis indicated that the 211F glands were slightly but significantly longer than their WT counterparts of the same age, and these differences were statistically significant (Fig. 1D). These surprising discoveries indicate a noteworthy advantage in gland development for the 211F mice, leading to enhanced growth of mammary glands compared to their WT counterparts. The augmented number of TEBs and the increased length of the glands in the 211F mice imply an elevated level of glandular development and growth, hinting at a potential beneficial effect associated with the 211F genotype in this context.

**Fig. 2:**
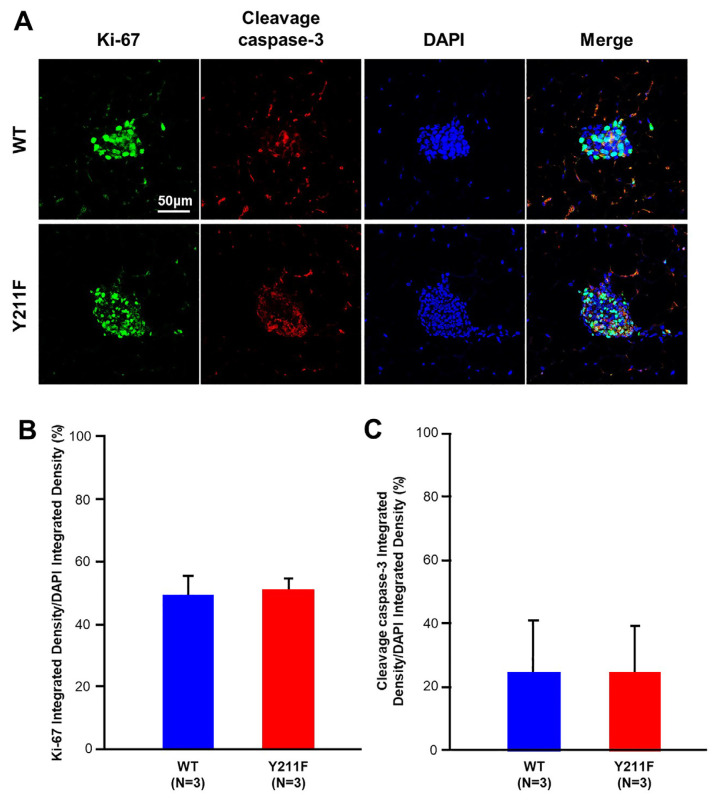
Y211 phosphorylation of PCNA did not alter local proliferation or cell death. (A) Immunofluorescence staining of Ki67 and the cleaved caspase-3 was performed on the mammary glands of 10-week-old WT and 211F FVB mice. Representative images of the immunofluorescence staining are shown. Scale bar, 50 μm. (B) and (C) The results in (A) Fluorescence images were quantified with ImageJ. Initialization and 8bit conversion preceded quantitative analysis. A defined threshold improved region delineation, and the Particle Analysis tool systematically quantified parameters area, intensity, and particle count. Statistical analysis was conducted using Student’s t-test. There was no significant difference between the WT and 211F glands.

The phosphorylation of Y211 on PCNA is recognized to promote cell proliferation in breast cancer cells [11,16]. To examine whether alterations in proliferation or cell death underlie the effects of Y211 phosphorylation on gland development, we stained mammary glands from WT and 211F mice for Ki67 (a proliferation marker [17]) and cleaved caspase 3 (an apoptosis marker [18]) (Fig. 2A). Notably, no discernible differences were observed in Ki67 expression or cleaved caspase 3 levels between the mammary glands of WT and 211F mutant mice (Fig. 2B and C). These results imply that the enhanced gland development in 211F mice is not driven by changes in local proliferation or cell death rates. Therefore, the increased gland growth observed in 211F glands is unlikely to be attributed to transient turnover of mammary epithelium.

**Fig. 3:**
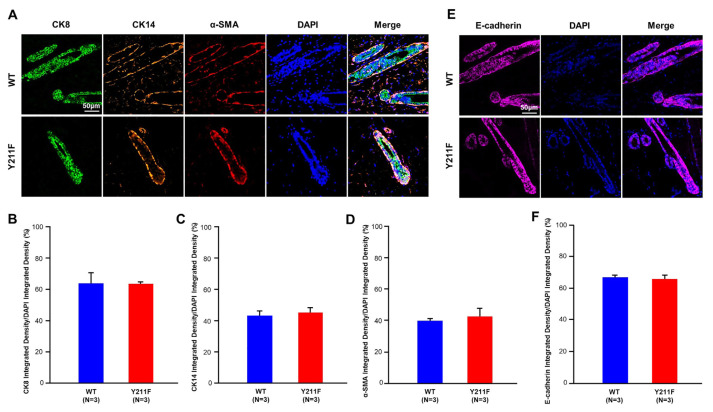
Assessment of mammary gland morphology and marker expression. The mammary gland tissues from 10-week-old WT and 211F FVB mice were stained by immunofluorescence with antibodies against the luminal epithelial marker cytokeratin-8 (CK8), basal myoepithelial marker cytokeratin-14 (CK14), alpha-smooth muscle actin (αSMA), and E-cadherin. (A) Representative images of the immunofluorescence staining are presented. Scale bar, 50 μm. (B–D) The expression levels of cytokeratin-8, cytokeratin-14, and αSMA within the mammary gland tissue were quantified using ImageJ. Statistical analysis was performed using Student’s t-test and there was no significant difference between the WT and 211F glands. (E) Representative images of the immunofluorescence staining of E-cadherin are shown. Scale bar, 50 μm. (F) E-cadherin expression in the mammary gland tissue was quantified using ImageJ. Statistical analysis was conducted using Student’s t-test. There was no significant difference between the WT and 211F glands.

Next, we explored other pivotal factors in mammary gland biology. E-cadherin, expressed in all normal mammary epithelial cells, plays a crucial role in promoting cell adhesion and the development of proper epithelial architecture [19]. Immunofluorescence staining of E-cadherin revealed no significant differences between the mammary glands of WT and 211F mutant mice (Fig. 3A and B). Additionally, to ascertain whether Y211 phosphorylation of PCNA could induce expansion of distinct cell origins, namely the luminal and basal epithelia of mammary glands, we assessed the expression of luminal epithelial marker (cytokeratin-8/CK8), basal myoepithelial marker (cytokeratin-14/CK14), smooth muscle alpha actin/αSMA) through immunofluorescence staining (Fig. 3C). In both WT and 211F glands, cytokeratin-8protein expression was localized to the luminal compartment, while cytokeratin-14 and αSMA markers were confined to the basal layer of the epithelium. Quantitative analysis of images demonstrated no significant differences in the expression levels of these markers between WT and 211F mutant mammary glands (Fig. 3, D–F). These results underscore that both WT and 211F glands have preserved the intricate morphogenetic structures of the mammary epithelium.

**Fig. 4:**
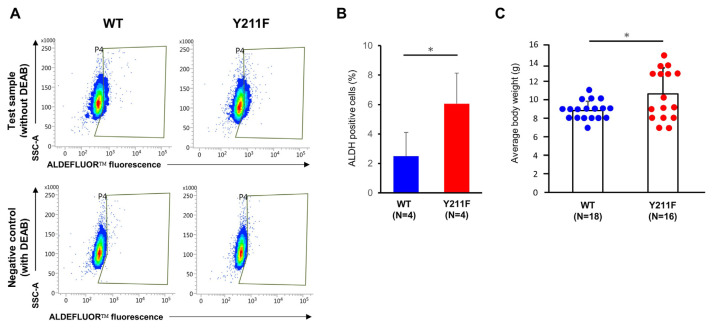
Increased ALDH-positive cells in the mammary gland and increased post-natal body weight in the 211F compared to the WT mice (A) Representative flow cytometry plots illustrating ALDH-positive cell populations in WT and 211F mammary glands. (B) Quantitated data are shown as means ± SD, and statistical significance was tested by Student’s t test, *, p < 0.05. (C) Average body weights of weaning animals at 3 weeks of age. Data are shown as means ± SD, and statistical significance was tested by Student’s t test, *, p < 0.05.

The enzyme activity of aldehyde dehydrogenase (ALDH) has been shown as a proficient biomarker for mammary progenitor cells across normal and cancerous tissues [20]. Within the normal breast epithelium, ALDH-positive cells represent a subpopulation with extensive lineage differentiation potential and remarkable regeneration capacity during duct regrowth [20]. Phosphorylation of PCNA at Y211 is associated with enhanced tumor stemness with higher levels of ALDH activity [15]. To gain insight into the ALDH activity of WT and 211F epithelial cells, primary mammary glands were dissected from both mouse types. Cells were then dissociated using enzymatic digestion and a dissociator (Miltenyi), followed by assessment of ALDH activity using the ALDEFLUOR assay. This assay allows analysis of cells with varying levels of ALDH activity through fluorescence-activated cell sorting (FACS). Remarkably, the results revealed significantly higher activity in the 211F epithelium compared to the WT epithelium (Fig. 4A and B). This indicates a potential enhancement in the progenitor capabilities within the 211F glands. The expansion of the stem cell compartment and the facilitated gland development suggest an enhancement of post-natal development. Indeed, a comparison between the body weights increase since weaning from the WT and 211F strains revealed that the 211F exhibited greater weight gains at waning compared to WT (Fig. 4C).

We then attempted to determine the potential originating lineage of stem cells responsible for the observed augmentation in stemness. In this regard, the basal myoepithelial cells found in mammary ducts, recognized as primary sources of bipotent mammary stem cells [4,7], can be identified using the mesenchymal-like stem cell surface markers CD24 and CD29. Specifically, CD24 + CD29+ is linked to increased potency, while CD24 + CD29− defines cells of luminal origin [21,22]. Interestingly, the proportions of CD24 + CD29+ and CD24 + CD29-cells were comparable in both WT and 211F mammary tissues (Supplementary Fig. 1). It should be pointed out that recent studies by others suggested that the basal myoepithelial cells remain bipotent postnatally until the disruption of the luminal-to-basal balance to trigger further lineage differentiation [6]. Therefore, the loss of Y211 phosphorylation on PCNA is associated with an increase in stem/progenitor potency likely in a yet-to-be-identified cell subpopulation.

Our investigation indicates that the absence of Y211 phosphorylation in PCNA enhances the normal growth of the mammary tree. This influence appears to be unrelated to cell turnover through proliferation or death. No discernible structural differences in epithelial composition were observed when comparing the WT and 211F glands. However, the population of aldehyde dehydrogenase-positive stem-like cells was elevated in the 211F mammary glands compared to the WT glands. Our data substantiate that reduced Y211 phosphorylation of PCNA can promote the development of mammary glands in mice by augmenting the pool of progenitor cells.

The finding that mammary glands with the 211F mutation exhibited a higher number of cells marked with stemness, coupled with increased growth compared to WT glands, appeared counterintuitive. This is because previous research has shown that in cancer cells, inhibiting Y211 phosphorylation of PCNA leads to reduced protein stability of PCNA on chromatin, thereby impeding cell proliferation [11]. The observed rise in the ALDH-positive population in the 211F glands, without concurrent changes in cell proliferation or death, suggests the existence of a previously unrecognized proliferation-independent mechanism in regulating mammary gland development, particularly within the eight to ten-week timeframe. One possibility is that the absence of Y211 phosphorylation may modulate cell division, subsequently slowing the early exhaustion of stem/progenitor cells and thereby contributing to the establishment of the stem cell niche for gland development. Further investigations are necessary to elucidate the underlying mechanism.

The present investigation underscores the distinct disparities between normal and cancer biology. Numerous previous studies have established that Y211 phosphorylation of PCNA plays a crucial role in promoting the proliferation of cancer cells [8,16]. However, in contrast, the elimination of Y211 phosphorylation did not exert an outstanding impact on the development of the 211F/PCNA mice [13]. This supports the notion that its function is largely dispensable in normal biology and suggests that targeting Y211 phosphorylation could be a potentially safe therapeutic strategy for cancer. The current study unveils an unforeseen consequence of abolishing Y211 phosphorylation of PCNA in the early development of mammary glands in virgin female mice under unstressed growth conditions. The potential enduring effects of pY211-PCNA in mammary gland turnover during pregnancy and the dioestrus cycles remain to be determined.

## Data Availability

The data generated and analyzed during the current study is available from the corresponding author upon reasonable request.

## Appendix

Supplement Fig. 1 The stemness of 211F glands is not derived from the mesenchymal-like stem cell niche. (A) Representative flow cytometry plots analyzing the expression of CD29 and CD24 in cells isolated from the WT and 211F mammary PCNA also plays a pivotal role gland. (B) Quantification of CD29^+^ and CD24^+^ subpopulation frequencies in WT and 211F mammary glands. Data represent mean ± S.D. (n = 3), and the statistical comparison with the WT group was assessed by the p values. There was no significant difference between the WT and 211F glands.
