# Supplementary figures and images for: Loss of tyrosine 211 phosphorylation of proliferating cell nuclear antigen (PCNA) enhances postnatal mammary gland development
Source: Biomedicine (Taipei). 2024 Sep 1;14(3):40–8. doi: 10.37796/2211-8039.1462 (PMC11460574; doi:10.37796/2211-8039.1462)

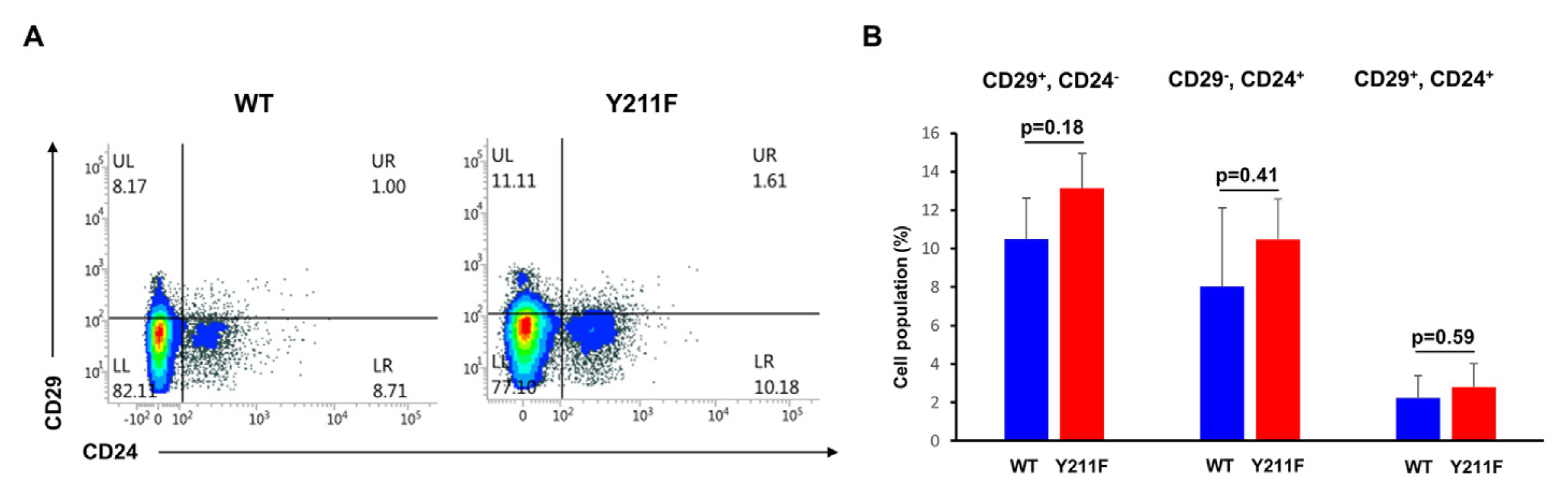

Supplement: Supplement Fig. 1 — The stemness of 211F glands is not derived from the mesenchymal-like stem cell niche. (A) Representative flow cytometry plots analyzing the expression of CD29 and CD24 in cells isolated from the WT and 211F mammary PCNA also plays a pivotal role gland. (B) Quantification of CD29+ and CD24+ subpopulation frequencies in WT and 211F mammary glands. Data represent mean ± S.D. (n = 3), and the statistical comparison with the WT group was assessed by the p values. There was no significant difference between the WT and 211F glands. [file bmed-14-03-040s1.tif]
